# The Effectiveness of Shoulder Mobility and Strength Programs in Competitive Water-Polo Players

**DOI:** 10.3390/life12050758

**Published:** 2022-05-20

**Authors:** Isaac López-Laval, Sebastian Sitko, Jaime Cantonero, Francisco Corbi, Rafel Cirer-Sastre

**Affiliations:** 1 Faculty of Health and Sport Sciences (FCSD), University of Zaragoza, 22002 Huesca, Spain; sitko@unizar.es (S.S.); 777017@unizar.es (J.C.); 2 Institut Nacional d’Educació Física de Catalunya (INEFC), Universitat de Lleida (UdL), 25192 Lleida, Spain; f@corbi.neoma.org (F.C.); rcirer@inefc.es (R.C.-S.)

**Keywords:** water-polo, range of movement, strength, acute effects

## Abstract

Background: Water-polo is the water sport with the highest incidence of injuries, with shoulder pain being the most common one. The understanding of risk factors and guidance on preventive measures is essential in this sport discipline. The aim of this study was to determine the effects of a specific 6-week training plan on range of motion (ROM) and joint strength levels in a group of professional water-polo players. Methods: Quasi-experimental study with a sample of 28 participants (age: 20.1 ± 2.5 years; height: 176.9 ± 6.2 cm; body mass: 74.6 ± 8.1 kg). Three study groups, which consisted of one control group and two experimental groups, were established. Two repeated measurements, pre and post intervention, were performed. During these measurements, ROM of the glenohumeral joint was analyzed both in external (ER) and internal (IR) rotation, as well as the maximal isometric strength. Conclusions: The application of a training program improved glenohumeral joint ROM. ER and IR evolve differently in both shoulders. ER improved only in the throwing arm only in the group undergoing intervention but for the non-dominant side, improvements were observed in both ER and IR, regardless of whether or not they had followed the intervention plan. No improvements were observed in either the isometric strength or contralateral asymmetries.

## 1. Introduction

Water-polo is a collaborative opposition sport that combines throwing actions, technical ball skills, explosive speed, and continuous swimming [1]. Physical contact between players and large training volumes result in water-polo being the aquatic sport with the highest incidence of injuries [2]. Injuries to the head and fingers resulting from blows and struggles between players, and overuse of the glenohumeral joint generated by constant repetitive movements during swimming and ball throwing, are the most common injuries [3]. Some authors found an injury incidence of 56.2 injuries per 1000 h of play in a competitive situation [4]. One of the peculiarities of this sport is that, like other disciplines such as baseball, volleyball, swimming, handball, or tennis, it is considered an “overhead sport” in which the repetitive and chronic movement of the arm above of the head causes wear and joint overuse [5]. The evidence has shown higher prevalence among overhead sports versus non-overhead sports (61% vs. 33%) [6]. Related to this, shoulder pain is the most common water-polo injury with a reported prevalence of around 24–80% [7]. In swimming, glenohumeral joint injuries may range from 40 to 91% depending on the age group and definition [8], whereas in handball and baseball, a high percentage of athletes reported shoulder pain in the early preseason. According to Wilk et al. (2020), glenohumeral joint problems were in the top four most commonly performed surgical procedures in overhead sports [9].

The main causes reported for this issue in relation with the overhead sports are the range of motion (ROM) and the relationship between joint strength deficit and external rotation (ER) to internal rotation (IR) ratio [10,11]. Water-polo players have increased ER and decreased IR combined with increased ROM at the dominant glenohumeral joint in comparison to its contralateral side [10,12]. Despite this, there is some controversy regarding the incidence of injury and ROM. For instance, Witvorow et al. (2003) concluded that a limited ROM in the glenohumeral joint has a direct relationship with the incidence of injury in overhead sports due to poor musculoskeletal predisposition to release high levels of elastic energy in high-speed and high-intensity movements [13]. Despite this claim, previous studies with professional water-polo players have not found a direct relationship between shoulder pain and limited ROM [14]. On the contrary, studies that differentially analyzed ROM in ER and IR established a relationship between injured players and a lower ROM in ER than in IR (*p* < 0.05). Along the same lines, Witwer and Sauners (2006) suggested that those water-polo players who did not suffer from pain in the glenohumeral joint presented an increased ER and reduced IR [10].

Other variables studied regarding the possible intra and inter-articular ROM asymmetries and their relationship with injuries are the “glenohumeral internal rotation deficit” (GIRD = decreased IR movement compared to the contralateral side), the “glenohumeral external rotation deficit” (GERD = decreased ER movement compared to the contralateral side), and the asymmetry between the total range of motion of each joint (_total_ROM = ER + IR of each arm). Despite the scarce scientific literature that addresses this variable and its incidence in the field of water-polo, it is known that a glenohumeral rotation deficit of more than 9.8° is associated with a possible shoulder injury. Regarding _total_ROM, it has been determined that a deficit greater than or equal to 7.5° is a predictor of future injuries and indicates contralateral decompensation [15].

Another important element that should be highlighted, not only because of its relationship with performance but also with injury prevention, is the strength of the glenohumeral joint-stabilizing groups. On the one hand, possible force imbalances in the rotator cuff are responsible for the braking during the throwing action. In these situations, a lack of IR strength implies a greater risk of injury in the deceleration phase [16]. On the other hand, the imbalances generated between forces in ER and IR (ER: IR) determine that despite the fact that there may be a difference in force production during ER and IR between dominant arm and non-dominant arms (ER = 133.1 N vs. 128.4 N and IR = 207.9 N vs. 196.8 N respectively), there are hardly any variations in the inter-articular ratio (ER:IR = 0.65 dominant arm vs. 0.67 non-dominant arm) [17]. Despite all this, imbalances in force production are synonymous with a greater probability of suffering an injury [18].

Understanding risk factors and targeting preventive measures are fundamental elements when attempting to improve ROM and joint force production in water-polo players [19]. Therefore, it was hypothesized that the use of specific protocols designed for this purpose could lead to more efficient ROM and improvements of strength in the glenohumeral joint. Accordingly, the aim of this study was to determine the effects of a specific 6-week training plan on ROM and joint strength levels in a group of professional water- polo players.

## 2. Materials and Methods

### 2.1. Research Design

The present study was quasi-experimental design with three convenience groups and two repeated measures of the outcome variables, one before the intervention plan training (T_Pre_) and the other one just after finishing the intervention (T_Post_). Twenty-eight participants completed this study to investigate the effects of a training plan on the glenohumeral joint ROM and the possible variations in the force applied by professional water-polo players. A first assessment was carried out the week before the start of the study (T_pre_), where joint mobility tests in both ER and IR and the maximum isometric force test in ER and IR were performed. All tests were performed with both the dominant and non-dominant arms. Once T_pre_ had concluded, the intervention program was applied 3 sessions per week (the plan details are displayed in Table 1.) After 6 weeks, a new assessment was carried out following the same order described (T_post_). Measurements were always made in the same place (University Lab, Río Isuela Sport Center, Huesca, Spain), with a mean temperature of 21 ± 2 °C and mean relative humidity of 52 ± 9%. Evaluations were carried out on Thursday and Friday afternoons (from 5 to 8 pm). Participants waited between 48 and 72 h before starting the intervention plan and standardized training after T_pre_, and also after the completion of the 6 weeks of work, before T_post_.

The study followed the ethical guidelines of the 2013 Declaration of Helsinki and received approval from the Research Ethics Committee of the autonomous region of Aragon, Spain (The approval code PI21/465, approved on 22 December 2021).

### 2.2. Participants

Thirty-one subjects voluntarily participated in this study. Twenty-two of the 31 participants were recruited from a professional water-polo team from the Spanish national first division and the rest were undergraduate Physical Activity and Sports Science students. The different groups in this study were: a group of water-polo players that performed the intervention plan (WI_group_; *n* = 11), a group of water-polo players that did not perform the intervention (W_group_; *n* = 10) and a control group (C_group_; *n* = 10). The water-polo players (*n* = 22) were randomly distributed into two different groups, WI_group_ and W_group_. Three subjects belonging to the group of water-polo players did not complete the whole study (*n* = 2 in WI_group_ and *n* = 1 in W_group_. Dropout rate = 9.6%). The main characteristics of the participants are reported in Table 2. During the intervention, the experimental group of water-polo players (WI_group_ and W_group_) followed the regular training with the team together and only the WI_group_ carried out the training plan before the regular practice. For C_group_, it was established that during the intervention period they could only perform the physical and practical activities that were part of their study plan. The water-polo players (WI_group_ and W_group_) were only allowed to perform their regular training sessions with their teams, except for the intervention program for the WI_group_. As inclusion criteria for the group of water-polo players, it was established that they should complete 4 training sessions per week plus competition matches and a minimum training volume of 9 h per week. Exclusion criteria were (i) did not have previous ailments or pathologies in the shoulder joint; (ii) did not have discomfort or pain in the shoulder that would result in the exclusion from the study; and finally, (iii) they were required not to have either used medications or drugs in a previous period of 6 months before the start of the study. After being informed of the benefits and potential risks of the investigation, all participants signed an informed consent.

### 2.3. Data Collection

All participants were weighed and measured during the T_pre_. Height (cm) was measured using the SECA-360 measuring rod (SECA©, Hamburgo, Germany) with a precision of 1 mm, and bodyweight (kg) was measured using scales of the same brand with a precision of 0.1 kg. Both tests, T_pre_ and T_post_, were carried out between 5 and 8 pm. Assessments were supervised by two liscensed physical activity specialists.

The protocol proposed by Hams et al. (2019) was used for the evaluation of shoulder ROM [14]. For this, the subject was placed on a stretcher in supine position, with the body completely supported on the stretcher and the shoulder placed at 90° abduction, elbow at 90° flexion, and forearm in a neutral position. Passive measurement of ROM in both ER and IR was performed with the TruMedical Baseline^®^ bubble 360° inclinometer. For the measurement of ER, the device was placed centered in the ventral side of the forearm, (2 cm from the proximal to the styloid process of the ulna). For IR, the device was placed on the dorsal surface of the forearm in the same arrangement as for ER. Scapular movements were not allowed. The dominant arm was assessed first, followed by the non-dominant arm for both ER and IR. Rotation was performed to the maximum limit of passive ROM. Shoulder elevation in regard to the position of contact with the stretcher or the subject’s perception of pain was used as the criteria to determine the maximum passive ROM value. Measurements were made in degrees of ROM. Three attempts were made per measurement and the mean value of the repetitions was determined as reference data.

For the measurement of isometric strength, the protocol used by Terol-Sanchis et al. (2021) was performed. The measurements were made with the participant seated on a chair, in a position that allowed him to stand with a 90° hip flexion, thighs resting on the seat and knees at 90°. The arm that was not measured was placed resting on the thigh of that same side. The arm under evaluation was placed in a position of 90° elbow flexion [20]. Measurements were made with a strain gauge (Chronojump Bosco System^®^, Barcelona, Spain), which has proven to be a reliable and valid measure to assess strength [21]. Before carrying out the measurements, the gauge was calibrated with the standardized weight recommended by the manufacturer. Three maximum voluntary contractions were performed for each arm both in ER and IR, and the maximum peak force obtained in N was noted. Each contraction was performed in such a way that no strange movements were produced with the body to avoid compensations that could increase the generated force value. Recovery between repetitions was 1 min.

### 2.4. Statistical Analysis

Data cleaning, manipulation, and analyses were performed in R (R Core Team 2021), and model parameters were described and reported according to bayestestR Reporting Guidelines. Descriptive data of participants were reported with mean (SD). Bayesian linear mixed-effects models were fitted to each variable as a function of dummy-coded factors time (reference level “T_pre_”), group (reference level “C_group_”), and their interaction, using the Stan modeling language (Stan Development Team 2021) and the package brms. The models included maximal random-effect structures justified by the non-randomized group allocation, allowing the predictors of interest and their interactions to vary by participants. Default priors of the brms package were used. Four sampling chains were run for 2000 iterations with a warm-up period of 500 iterations for each model, thereby yielding a total of 6000 post-warmup draws. For all relevant cell means and differences between them, the expected median values under the posterior distribution and their 95% credible intervals (CIs) were reported. To describe the existence of each effect, the probability of direction (pd), which represents the certainty associated with the most probable direction (positive or negative) of an effect, was reported. According to Makowski et al. (2019), pd ≤ 95%, pd > 95%, pd > 97%, pd > 99%, and pd > 99.9% were reported as uncertain, possibly existing, likely existing, probably existing, and certainly existing, respectively [22]. 

## 3. Results

**Figure 1:**
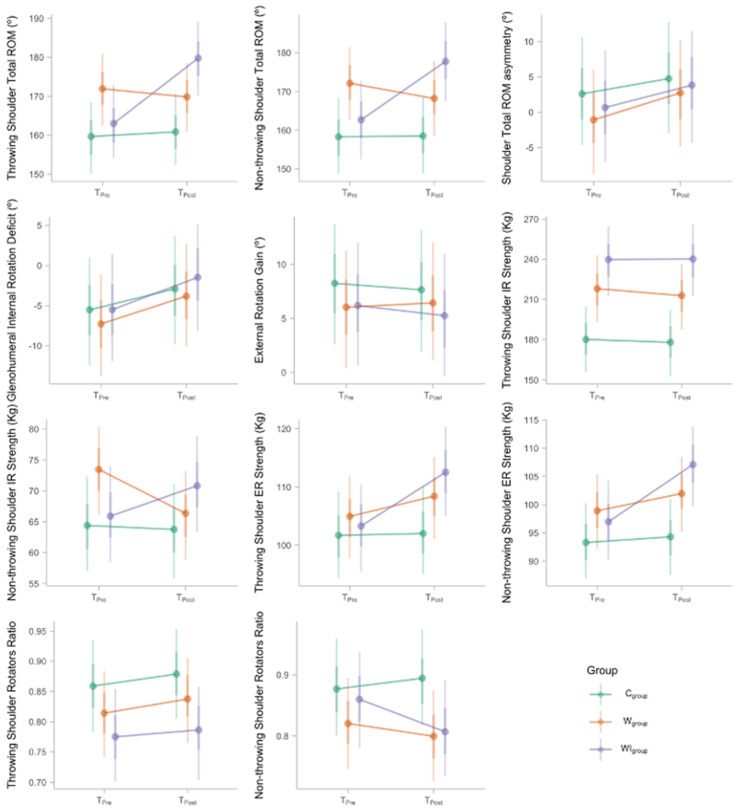
Time effects by group (color) and variable (subplot). Dotted, heavy, and light vertical lines indicate the median, 95% CI, and range of the model posterior distributions, respectively.

A summary of model posterior distributions can be found in Table 3. Adding a training program to regular water-polo sessions resulted in a significant improvement in the ROM of both shoulders (dominant, median increase = 16.73°, 95% CI from 11.56° to 22.07°, pd = 100%; non-dominant, median increase = 15.29°, 95% CI from 8.15° to 22.56°, pd = 99.97%). Meanwhile, changes in ROM were negligible in the W_group_ (dominant, pd = 79.52%; non-dominant, pd = 87.52%) and C_group_ (dominant, pd = 67.78%; non-dominant, pd = 51.45%). Despite its effect on total ROM, the training program did not influence _total_ROM (median change = 3.08°, 95% CI from −5.92° to 12.08°, pd = 75.03%), GIRD (median change 2.08°, 95% CI from −5.28° to 9.43°, pd = 71.38%), or GERD (median change = −0.93°, 95% CI from −4.98° to 3.29°, pd = 66.83%). The same findings apply also to the W_group_ and C_group_ (Figure 1).

Rotation torque, on the other hand, evolved differently between shoulders. In the throwing shoulder, IR torque did not improve in any group (WI_group_ median change = 0.31°, 95% CI from −14.31°, pd = 51.87%; W_group_ median change = −5.12°, 95% CI from −19.03° to 7.75°, pd = 78.73%; C_group_ median change = −2.09°, 95% CI from −15.11° to 10.70°, pd = 62.07%). By contrast, ER torque improved in the players who performed the training program (WI_group_ median change = 9.30°, 95% CI from 4.60° to 14.01°, pd = 99.97%), but not much in the W_group_ (median change = 3.49°, 95% CI from −1.12° to 7.91°, pd = 93.37%) and not at all in the C_group_ (median change = 0.32°, 95% CI from −4.25° to 5.04°, pd = 55.52%). For the non-throwing shoulder, IR torque improved for the WI_group_ (median change = 4.88°, 95% CI from −0.12° to 9.70°, pd = 97.27%) but decreased in the W_group_ one (median change = −7.08°, 95% CI from –12.03° to −2.49°, pd = 99.67%), without meaningful changes in the C_group_ (median change = −0.64°, 95% CI from −5.75° to 4.32°, pd = 60.22%). In addition, non-throwing shoulder ER torque improved only in the WI_group_ (median change = 10.21°, 95% CI from 5.60° to 14.57°, pd = 100%), but not clearly in the W_group_ (median change = 3.02°, 95% CI from −1.10° to 7.64°, pd = 91.33%) and not at all in the C_group_ (median change (0.95°, 95% CI from −3.55° to 5.52°, pd = 65.95%).

Rotation ratios of strength did not change over time in any group, neither for the throwing shoulder (WI_group_ median change = 0.01, 95% CI from −0.05 to 0.08, pd = 62.22%; W_group_ median change = 0.02, 95% CI from −0.04 to 0.08, pd = 78.65%; C_group_ median change = 0.02, 95% CI from −0.05 to 0.08, pd = 73.20%), nor for the non-throwing one (WI_group_ median change = −0.05, 95% CI from −0.13 to 0.01, pd = 93.88%; W_group_ median change = −0.02, 95% CI from −0.09 to 0.05, pd = 73.32%; C_group_ median change = 0.02, 95% CI from −0.05 to 0.09, pd = 69.52%).

## 4. Discussion

The results of this study suggest that a 6-week training program improves the ROM of the glenohumeral joint of the WI_group_ in relation to W_group_ and C_group_, without modifications in the comparisons of contralateral asymmetry between shoulders. The relative values of ROM in ER and IR evolved differently. For the dominant side (throwing arm), ER improved only in the group of water-polo players following the intervention program (WI_group_). However, this was not observed in any group for the IR assessment. Regarding the non-dominant side, improvements were observed in both ER and IR in WI_group_ and W_group_ but not for the C_group_. Lastly, no improvements in strength were observed in any of the three groups.

From a global perspective, our results are comparable to previous studies that reported improvements in the ROM of the glenohumeral joint after the implementation of training plans [7,10,23]. These data support the idea of applying a program parallel to normalized training to improve tissue elasticity of the shoulder joint. According to Yanai et al. (2000), mobility and stretching exercise must be included in the training plan and are adequately completed to the regular training week [24]. The possible asymmetries between arms, on the other hand, were comparable between the groups for the GIRD, GERD, and _total_ROM variables both in the T_pre_ and T_post_ situations. In addition, despite identifying an improvement in ROM in the WI_group_, no changes in the asymmetry between the dominant and non-dominant arms were observed, and the values obtained were always within the expectable variations accepted as a possible risk of injury; less than 9.8° and 7.5° for the glenohumeral joint in rotation deficit and _total_ROM, respectively [15]. We should also highlight that previous studies with other overhead sports (baseball) showed a statistical relationship between injury risk and asymmetries that is lower than those reported in our work (<5°) [25]. Despite this, our results align with those of Borsa et al. (2008), who stated that ROM should be the same in both shoulders in athletes who perform overhead sports [26].

Considering the movement peculiarities of this sport and the results obtained in this study, different biomechanical requirements are proposed for each side of the body. On the one hand, the throwing arm demands high mobility to guarantee muscle activation and relaxation in the dynamic explosive actions typical of the throwing action. On the other hand, the contralateral side is mainly subject to the swimming action and passing the ball. In terms of ER improvement of the throwing arm, the application of a specific work plan confirmed the descriptive values for water-polo players. The ER improvement in the throwing arm after the application of a specific work plan confirmed the descriptive values for water-polo players and other overhead sports such as swimming [27], handball [28], tennis, and baseball [29], which showed similar ER values in the dominant arm. The fact that ROM improvements are achieved in the ER is in line with the relationship between the lower incidence of injuries and a greater ROM in ER [10,14], but would also help to improve performance in the throwing action. Despite performance indicators not being evaluated in this study, some studies have found improvements in ER with the increase in the range during the loading phase, which leads to better performance due to a larger ROM available. Though throwing speed was not evaluated in this study, this situation could translate into a greater distance in the acceleration phase and therefore an evident increase in the speed of the throwing action [10,16]. The lack of IR improvement in the intervention groups (both in WI_group_ and W_group_) might be due to the fact that this mobility is associated with reactive scarring or chronic contracture of the periscapular soft tissues as a consequence of the continuous braking phases typical of the throws performed in this sport [26]. This theory could also explain why improvements in IR of the dominant arm were not obtained neither for the group of water-polo players submitted to the intervention plan (WI_group_) nor for the group of water-polo players who followed standardized training (W_group_).

The results obtained for the non-dominant arm coincide with the work by Hams et al. (2019), who stated that repetitive movements during normalized training in the non-dominant arm are sufficient stimulus to generate musculoskeletal adaptations [15]. This would explain why improvements have been obtained in both intervention groups. Therefore, and regarding these data, we question the need to apply specific training plans for the non-dominant side if the athlete has already acquired normal ROM values. It seems that water-polo standardized training is associated with gains in both ER and IR in this joint.

Regarding the strength variable, previous studies have shown a relationship between higher injury risk in the glenohumeral joint and ER weakness in overhead sports [30,31]. Therefore, it was assumed that carrying out an additional training program would significantly improve both ER and IR strength values in the WI_group_, however, no differences were found between groups. This suggests that the prescribed intensity and exercise execution speed did not provide enough stimulus to achieve specific strength adaptations [32]. Although evidence of strength improvements from a performance perspective exists in semi-professional and professional water-polo players [33,34], the results of this study are similar to those obtained in other investigations that followed similar training plans and where no improvements in strength were found from a functional point of view [22,35]. Our results are comparable to Swanik et al. (2002), who reported no significant strength differences between study groups after a 6-week functional training program that included rubber-tubing, dumb-bell, and body-weight exercises [36]. When analyzing the scientific literature, contradictory and varying effects can be found. Some studies highlighted the efficiency of carrying out training programs parallel to normalized training, while others did not find improvements [37,38]. Therefore, it should be considered that the type of intervention designed by the coaches has a direct relationship with the training effect that is intended to be achieved and that applying specific training does not guarantee an injury risk reduction or a performance improvement. Despite this, the general recommendations regarding overhead sports determine the need to apply functional-type exercises that improve the levels of rotational strength of the shoulder, especially in the ER phase of the joint [39]. According to a recent review, the true efficiency of training protocols to limit shoulder injuries in overhead athletes must be further investigated [40].

There were several limitations in the current study. Previous research assessed short-term training adaptations after intervention programs. Given the context of this study and the sample that was recruited (professional water-polo players), it was impossible to propose a study of these characteristics. Further, future studies should consider specific performance factors related to this sport discipline such as throwing or swimming speed. Finally, statistical modeling in this study was performed from a Bayesian framework, which has some advantages over the null-hypothesis significance testing. This leads to the fact that our results have clear and valid interpretation, regardless of the number of observations provided [41]. Although this implies that a minimal sample size is not needed for a model to be valid, larger and randomized groups would have certainly improved the quality of our results.

## 5. Conclusions

A specific mobility and strength training program produced improvements in the ROM of the glenohumeral joint. ER and IR evolve differently in both shoulders, with ER improving in the throwing arm only in the group of water-polo players undergoing intervention, but not in any other group in the IR assessment. For the non-dominant side, improvements were observed in both ER and IR in all water-polo players, regardless of whether or not they had followed the intervention plan. No improvements were observed in either the strength variable or possible contralateral asymmetries. Future lines of research should analyze the designed training plan itself and be based exclusively on the most effective methods both for reducing the risk of injury and for improving performance. 

## Figures and Tables

**Table 1:** Characteristics of the intervention strengthening program.

Weeks and Days	Sets × Reps	Exercises
6 weeks 3 days per week	3 × 15	Shoulder flexion; shoulder extension; IR at 90°; ER at 90°; throwing acceleration; throwing deceleration; low rows; scapular punches; Y’s; T´s; W´s
**Exercise**	**Muscular activity**	**Characteristic addressed**
Shoulder flexion	DA, Rhomb, SA, Sub, RM	Strengthen scapular stabilizers
Shoulder extension	Dors, Rhomb, Sub, Tri, RM	
IR at 90°	TI, Rhomb, SA, Sub, RM	
ER at 90°	TI, Rhomb, SA, Sub, Supra, RM	Strengthen scapular stabilizers, weak ER
Throwing acceleration	TI, Rhomb, SA, Sub, RM	Strengthen scapular stabilizers, improve propioception
Throwing decceleration	TI, Rhomb, Sub, Supra, RM, TS	Weak ER, improve propioception
Low rows	Rhomb, Sub. RM	Strengthen scapular stabilizers
Scapular punches	Rhomb, SA, Sub, RM	Strengthen SA
Y arms movement	TI, TM, SA	Strengthen scapular stabilizers, increases scapular up rotation, post tilt, retraction, and ER
T arms movement	Infra, TM, SA, RM, TS	
W arms movement	Infra, TI, Rhomb, Supra, RM	

Abbreviatons: DA, anterior deltoid; Rhomb, rhomboides; SA, serratus anterior; Sub, subescapularis; RM, teres minor; Dors, latissimus dorsi; Tri, triceps; Supra, supraespinatus; TI, lower trap; TM, middle trap; TS; upper trapezius; Infra, infraespinatus.

**Table 2:** Summary of the participant characteristics.

	C_group_ (*n* = 10)	W_group_ (*n* = 9)	WI_group_ (*n* = 9)	Overall (*n* = 28)
Age (y)	22.2 ± 1.2	18.6 ± 2.4	19.8 ± 2.5	20.1 ± 2.5
Height (cm)	176.7 ± 5.3	177.2 ± 3.7	178. 7 ± 6.8	176.9 ± 6.2
Body mass (kg)	72.2 ± 5.1	75.1 ± 7.7	74.4 ± 7.8	74.6 ± 8.1

**Table 3:** Summary of results.

		C_group_ (*n* = 10)	W_group_ (*n* = 9)	WI_group_ (*n* = 9)
Shoulder Range of Motion (°).				
Throwing shoulder Total ROM	T_Pre_	160 (150–169)	172 (163–181)	163 (154–173)
	T_Post_	161 (152–171)	170 (161–179)	180 (170–189)
Non-throwing shoulder Total ROM	T_Pre_	158 (149–168)	172 (163–181)	163 (152–173)
	T_Post_	157 (149–169)	167 (159–178)	178 (167–188)
Total ROM asymmetry	T_Pre_	3 (−5–11)	−1 (−9–6)	0 (−7–9)
	T_Post_	5 (−3–13)	3 (−5–10)	4 (−4–11)
GIRD	T_Pre_	−5 (−12–1)	−7 (−14–−1)	−6 (−12–1)
	T_Post_	−3 (−10–4)	−4 (−10–3)	−2 (−8–5)
ERG	T_Pre_	9 (3–14)	6 (0–11)	6 (1–12)
	T_Post_	8 (2–13)	6 (1–12)	5 (0–11)
Shoulder Strength (kg)				
Throwing shoulder IR strength	T_Pre_	181 (156–204)	218 (193–243)	241 (212–265)
	T_Post_	180 (153–202)	212 (187–237)	239 (212–266)
Non-throwing shoulder IR strength	T_Pre_	64 (57–72)	73 (66–80)	66 (58–74)
	T_Post_	63 (56–71)	66 (59–73)	71 (63–79)
Throwing shoulder ER strength	T_Pre_	102 (94–109)	105 (98–112)	103 (95–110)
	T_Post_	102 (95–110)	108 (101–115)	113 (105–120)
Non-throwing shoulder ER strength	T_Pre_	93 (87–100)	99 (92–105)	96 (90–104)
	T_Post_	95 (88–101)	102 (95–108)	107 (100–114)
Throwing shoulder Rotators Ratio	T_Pre_	0.86 (0.78–0.94)	0.81 (0.74–0.88)	0.78 (0.70–0.85)
	T_Post_	0.88 (0.80–0.95)	0.83 (0.76–0.91)	0.79 (0.70–0.86)
Non-throwing shoulder Rotators Ratio	T_Pre_	0.87 (0.80–0.96)	0.82 (0.75–0.89)	0.86 (0.78–0.94)
	T_Post_	0.90 (0.82–0.98)	0.80 (0.73–0.88)	0.81 (0.73–0.89)

Abbreviations: ROM, range of motion; GIRD, glenohumeral internal rotation deficit; ERG, external rotation gain; IR, internal rotation; ER, external rotation; C_group_, control group; WI_group_, group of water-polo players that carried out the intervention plan; W_group_, group of water-polo players that did not carry it out. Note: Values are reported as the expected median (95% credibility interval) based on the model’s posterior distribution.
